# Supporting DACA recipients and international students pursuing careers in medicine: A pilot program for advisors

**DOI:** 10.1371/journal.pone.0281540

**Published:** 2023-02-06

**Authors:** Yoshiko Iwai, Keny Murillo Brizuela, Jesus Ruiz, Erin Gustafson, Mark G. Kuczewski, Gary L. Beck Dallaghan

**Affiliations:** 1 University of North Carolina at Chapel Hill School of Medicine, Chapel Hill, North Carolina, United States of America; 2 Department of Family Medicine, University of North Carolina at Chapel Hill School of Medicine, Chapel Hill, North Carolina, United States of America; 3 Office of International Students & Scholars, Yale University, New Haven, Connecticut, United States of America; 4 Neiswanger Institute for Bioethics, Loyola University Chicago Stritch School of Medicine, Chicago, Illinois, United States of America; 5 Office of Medical Education, University of North Carolina at Chapel Hill School of Medicine, Chapel Hill, North Carolina, United States of America; St John’s University, UNITED STATES

## Abstract

The United States (U.S.) health professions are becoming more invested in diversity. Information on students who are undocumented or recipients of Deferred Action for Childhood Arrivals (DACA), and international students on student visas entering U.S. medical education is sparse. Few programs offer targeted training for educators on advising students who are undocumented, DACA recipients, or on a visa. We piloted a virtual program for pre-health advisors and educators on supporting students who are undocumented or recipients of DACA and international students transitioning to medical school. Program evaluation consisted of an anonymous retrospective pre-post survey. Of 117 registrants, 40% completed the survey. Prior to the program, most participants indicated that they were unsure or thought students were ineligible for financial aid during medical school if they were DACA recipients (40% unsure, 26.6% ineligible) or on a student visa (30% unsure, 30% ineligible). After the program, most respondents reported students were eligible for merit scholarship or private loans with DACA (66.6% eligible) or an international student visa (60% eligible). Perceptions of students with DACA being able to lawfully practice medicine in the U.S. changed from pre-program (43.3% unsure or not eligible) to post-program (90% eligible). Participants indicated they were more confident advising DACA recipients and international students post program. This virtual program was an effective step in providing support for advisors who are assisting non-citizen or permanent resident students start their careers in healthcare. Our findings show the need for more information on advising students who are DACA recipients, undocumented, or on student visas prior to matriculating to medical school and throughout training.

## Introduction

As of 2016, approximately 30% of physicians in the United States (U.S.) were non-U.S. born and 7% were non-citizens [1]. Based on the Association of American Medical Colleges (AAMC) report of Deferred Action for Childhood Arrivals (DACA), students with DACA are anticipated to make up 5,400 to 31,860 of minority U.S. physicians in future decades [2]. In addition to students with DACA, international medical graduates comprise 24.5% of practicing physicians [3], and international students who graduate from U.S. medical schools (UMG) make up approximately 3% of applicants and 1% of matriculants [4, 5].

Immigrant physicians fill a critical role, caring for underserved communities and contributing to the racial, ethnic, and cultural diversity of U.S. healthcare [6]. Yet, previously undocumented minors protected under the DACA program and international students on visas who are training in the U.S. face significant barriers [7, 8]. Common obstacles include biases against race or immigration status, daily hurdles like not being able to obtain a driver’s license or financial aid, restrictions on employment and in the residency match, lack of social or familial support, and the constant emotional toll of an uncertain legal status [9–13]. These students are also often limited by the scarcity of programs that consider their applications [14], and frequently face financial, legal, institutional, and personal challenges in their journey to and beyond medical school [15, 16]. Despite these barriers, there is little targeted support for helping students in their undergraduate, medical education, and professional path.

Information on the experiences and outcomes of international UMGs and students with DACA pursuing careers in medicine is sparse. Knowledge gaps include the admissions process to medical school, transition to residency, and future practice in the U.S. under immigration restrictions [2, 17]. These gaps in information have led to the formation of organizations like F-1 Doctors [17], a peer-founded and led program for international students at U.S. medical schools and residencies, and Pre-Health Dreamers [18], a California-based organization offering guidance to students and educators on being undocumented and pursuing a career in healthcare. While these organizations typically offer mentorship on a one-to-one basis, there are limited resources at the institutional level.

Past programming efforts for students who are undocumented, DACA recipients, or on a student visa have primarily focused on providing information to learners [19, 20], despite gaps in knowledge among medical educators and administration [21, 22]. Thus, we saw an opportunity for targeting individuals who advise students who are undocumented, DACA recipients, or on a visa. The transition to virtual formats due to the Coronavirus Disease 2019 (COVID-19) pandemic and surge in efforts to diversify healthcare presented an additional opportunity. We developed a virtual program for pre-health advisors and educators to mentor DACA recipients and international students on their transition to medical school and better prepare them for a career in medicine.

## Materials and methods

This study was reviewed by the University of North Carolina Institutional Review Board (#21–0918) and determined exempt; thus, informed consent was not obtained. However, participants were informed of the possible risks, benefits, and intent of the study through a standardized information form.

The program was developed from November 2020 to June 2021 by two medical students (YI, KMB) who experienced challenges associated with being an international student and DACA recipient at a medical school in the U.S. The program was a voluntary two-day event for pre-health advisors and educators (including post-baccalaureate programs, graduate schools, and medical school advisors serving in pipeline programs). The first day focused on international students on an F-1 student visa and the second day on students with DACA or who are undocumented. The goals of this program were to: (1) assess current knowledge on DACA recipients and international students pursuing a career in medicine in the U.S.; (2) provide basic information on immigration regulations associated with DACA and international student statuses; (3) offer current medical student, resident, and physician perspectives on being a DACA recipient or international student in the U.S.; and (4) evaluate advisors’ confidence in supporting students through the transition into medical training before and after the program.

### Program content

The two-day program took place over a videoconferencing webinar (Zoom Video Communications, Inc., San Jose, California) in July 2021. The outline of the program is represented in Table 1. The speakers were recruited to participate in the workshop via email and a subsequent videoconferencing meeting was set up to discuss the program structure and expectations for each speaker. All presenters who were contacted agreed to participate. An honorarium was provided to the speakers after their participation in the program.

**Table 1:** Program overview on supporting DACA and international students in medicine.

Session	Topic	Presenter(s)	Duration	Learning Objectives
International Student	Introduction to entering medicine as an F1 student in the U.S.	• 1 Academic Admissions Consultant	45 minutes	• Discuss common challenges associated with transitioning to medical school on an F1 student visa • - Discuss recommendations and strategies for pursuing medical education in the U.S. on a visa
International Student	Fundamentals of immigration regulations for F1 students in medical school and residency	• 1 Designated School Official and International Scholar Advisor	45 minutes	• Discuss F1 visa restrictions related to medical school, residency applications, and employment • Discuss F1 visa transition process to residency in the U.S. • Discuss strategies for advocating for students throughout medical education
International Student	Panel with F1 medical students, resident physicians, DSOs	• 5 MD students • 1 MD/PhD student • 1 resident physician • 2 DSO (1 private, 1 public medical school)	60 minutes	• Introductions and path to medicine (including current immigration status and institution) • Discuss challenges throughout medical school admissions and residency application • Discuss things you wish you knew earlier in medical education
International Student	Resources for international students	• F1-Doctors student representatives	20 minutes	• Introduce F1-Doctors organization • Discuss peer mentorship benefits for international students and how educators can refer their students
DACA Student	Introduction to entering medicine as a DACA student in the U.S.	• 1 Chair of DREAMer committee and medical school faculty • 1 DACA recipient MD student	60 minutes	• Discuss common challenges associated with transitioning to medical school as a DACA student • Discuss recommendations and strategies for medical education in the U.S. as a DACA or DREAMer student
DACA Student	From Undocumented to Doctor	• 1 attending physician	45 minutes	• Discuss path to medicine as a formerly undocumented medical student, now physician • Discuss challenges and recommendations for advisors and educators to better support DACA students
DACA Student	Panel with DACA, medical students and physicians	• 3 MD students • 1 attending physician • 1 DACA student advisor	45 minutes	• Introductions and path to medicine (including current immigration status and institution) • Discuss challenges throughout medical school admissions and residency application Discuss things you wish you knew earlier in medical education
DACA Student	Resources for DACA students and advisors	1 Pre-Health Dreamers Executive Director	20 minutes	• Introduce Pre-Health Dreamers • Discuss peer mentorship benefits for DACA students and how educators can refer students • Discuss recommendations for programs to become more “DACA friendly”

Abbreviations: “DACA” indicates Deferred Action for Childhood Arrivals, an exercise of prosecutorial discretion for certain people who came to the U.S. as children and met criteria for age, timing, and physical residence when the order went into effect [7]. In August 2022, the Department of Homeland Security published the “DACA Final Rule” to fortify the DACA policy [7]. “DREAMer” indicates individuals who qualify for the Development, Relief, and Education for Alien Minors (DREAM) Act, which would protect certain immigrants who arrived in the U.S. as children by providing a path to legal status [8]. Despite bipartisan support of each version of the proposal, the DREAM Act is still only a proposed bill and has not yet become a law [8].

The first day consisted of an introduction to applying to medical school on an F1 visa, the most common visa category reserved for academic students enrolled in colleges, universities, and other academic institutions in the U.S., including health professional schools. This introduction was followed by a lecture on basic immigration regulations for F1 students applying to medical school and residency given by a Designated School Official (DSO) at a U.S. medical school who interfaces with the Student and Exchange Visitor Program within Immigration and Customs Enforcement (ICE). During this session, the presenter shared common dos and don’ts, for example, avoiding asking students when they will become U.S. citizens given permanent residency (i.e., obtaining a green card) must precede citizenship. Common paths to permanent residency were also discussed, including marriage to a U.S. citizen and employment, both of which often require extensive legal support. Additionally, F1 limitations on employment during and after studies, the challenges of finding visa sponsorship during residency, and the inability to accept federal financial aid were also discussed.

This was followed by a panel with medical students on F1 visas, a resident physician formerly on an F1 visa, and two DSOs (one from a state medical school and one from a private medical school) who serve as advisors to and communication channels between international students and ICE to help students acquire and maintain legal status during their studies. Panelists discussed their path to medical school or residency, unique barriers they faced, and advice that they wish they had received earlier in their medical training. Common anecdotes they shared included identifying gap year opportunities, negotiating scholarships, and the psychosocial pressures of putting together a competitive medical school application given the limited pool of programs that consider international student applicants. Program participants could submit questions to panelists which were discussed in real-time. The first day of the program concluded with a session from F-1 Doctors representatives who introduced the peer-support resource.

The second day began with an introduction to applying to medical school as a DACA recipient student, where an administrative leader and medical school faculty member shared general guidelines alongside his medical school’s requirements for admission. This talk was joined by a medical student who supplemented with his personal anecdotes on how he navigated the application process as a DACA recipient. The next session was from a current physician in the U.S. who was formerly undocumented. This physician shared his story from entering the country as an undocumented youth through his complex career path which led him to become a family medicine doctor serving many immigrant and underserved communities.

These sessions were followed by a panel including medical students who were undocumented and became DACA recipients, an attending physician who was formerly undocumented, and a Pre-Health Dreamers (PHD) program coordinator who advises undocumented and DACA recipient students through health professions careers. During the panel, the speakers shared anecdotes of finding scholarships to pay for college and medical school, creative ways for managing finances, and identifying mentors who shared similar backgrounds. The panel followed the same structure as the first day and participants could ask questions which were answered in real-time. The program concluded with a session from the PHD executive director who discussed resources on becoming “DACA-friendly” institutions and participating in local advocacy work. She shared PHD’s resources for leadership training and how to approach university administration, especially around redistributing scholarship funds. This session also included materials on “undocumented-friendly” programs to broaden support for students of various statuses.

All participants had access to recordings and resources that were shared during the sessions. Student, resident, and physician panels were not recorded to protect speaker privacy.

### Participant recruitment

Information on the program was shared through the National Association of Advisors for the Health Professions (NAAHP) listserv and direct emails to pre-health and post-baccalaureate program advising offices at U.S. colleges and universities. A standardized email script was used to advertise the program. This was a voluntary program and recruitment was based on personal or professional interest. NAAHP sent a reminder email to listserv subscribers before the program start date.

### Survey development and distribution

We developed one retrospective pre-post survey (S1 Appendix). Survey questions were created through multiple discussions and iterative edits by the research team, which included individuals with content expertise (JR, EG, MGK) and individuals with personal experience of undocumented, DACA, and student visa statuses (YI, KMB, JR). Survey questions assessed knowledge of DACA and international student barriers (including number of eligible medical schools, access to financial aid, and ability to practice medicine in the U.S. legally) and their confidence in supporting students with DACA and international visas before and after the program. Applicants rated statements on a 5-point Likert scale and had the opportunity to provide qualitative feedback (S1 Appendix). One author (GLBD) with survey research expertise reviewed the survey instrument for clarity and content. We did not pilot test the survey due to the limited study population.

The survey was sent to all participants one hour after the program. Two additional reminders were sent at one-week intervals following the program. No identifiable information was collected. Participants had the option to be entered into a drawing to receive a $50 or $100 gift card after completing the survey, and email addresses were stored separately from the data for these respondents. There was no cost associated with the program and participants were not compensated for their participation. The data were collected and analyzed anonymously.

### Data analysis

Numeric data from the survey was analyzed using descriptive and inferential statistics. Wilcoxon signed rank tests were used to compare responses, which were considered statistically significant if p < .05. Analyses were conducted using IBM SPSS version 28 (Armonk, NY). Open-ended survey responses were analyzed through direct content analysis, supplemented by open and axial coding [23]. One member of the research team (YI) reviewed all open response answers and summarized quotes that meaningfully described participant experiences or knowledge related to the program. These quotes were then reviewed by the research team to determine common themes. A final table with illustrative quotes and overarching themes was determined through discussion. Two members of the research team had qualitative expertise (YI, GLBD). An encrypted spreadsheet was used for all free text analysis (Microsoft Excel, 2019, Version 16, Redmond, WA: Microsoft Corporation).

## Results

Of 117 registrants, 71 attended the international student session, 40 attended the DACA session; 36 attended both live sessions resulting in 75 total unique participants. Registrants had the option of watching recordings on their own time. Of the 75 people who attended live, 30 (40%) completed the survey. Fifteen (50%) were pre-med or pre-health college advisors, 3 (10%) held leadership roles in college advising services, and 6 (20%) reported Other for their role. There was an average of 8.47 years in advising (range (SD), 0.0–36 (7.84) years). Among the respondents, 23 (76.7%) worked at public institutions and 7 (23.3%) at private institutions, with 23 (76.7%) at universities and 3 (10%) at liberal arts (private undergraduate programs with a broad-based curriculum) colleges. Participants joined from the following 14 states: Washington, North Dakota, Texas, Iowa, Illinois, Michigan, Wisconsin, New York, Connecticut, North Carolina, South Carolina, Georgia, Florida, and the District of Columbia.

### Survey results

**Fig 1:**
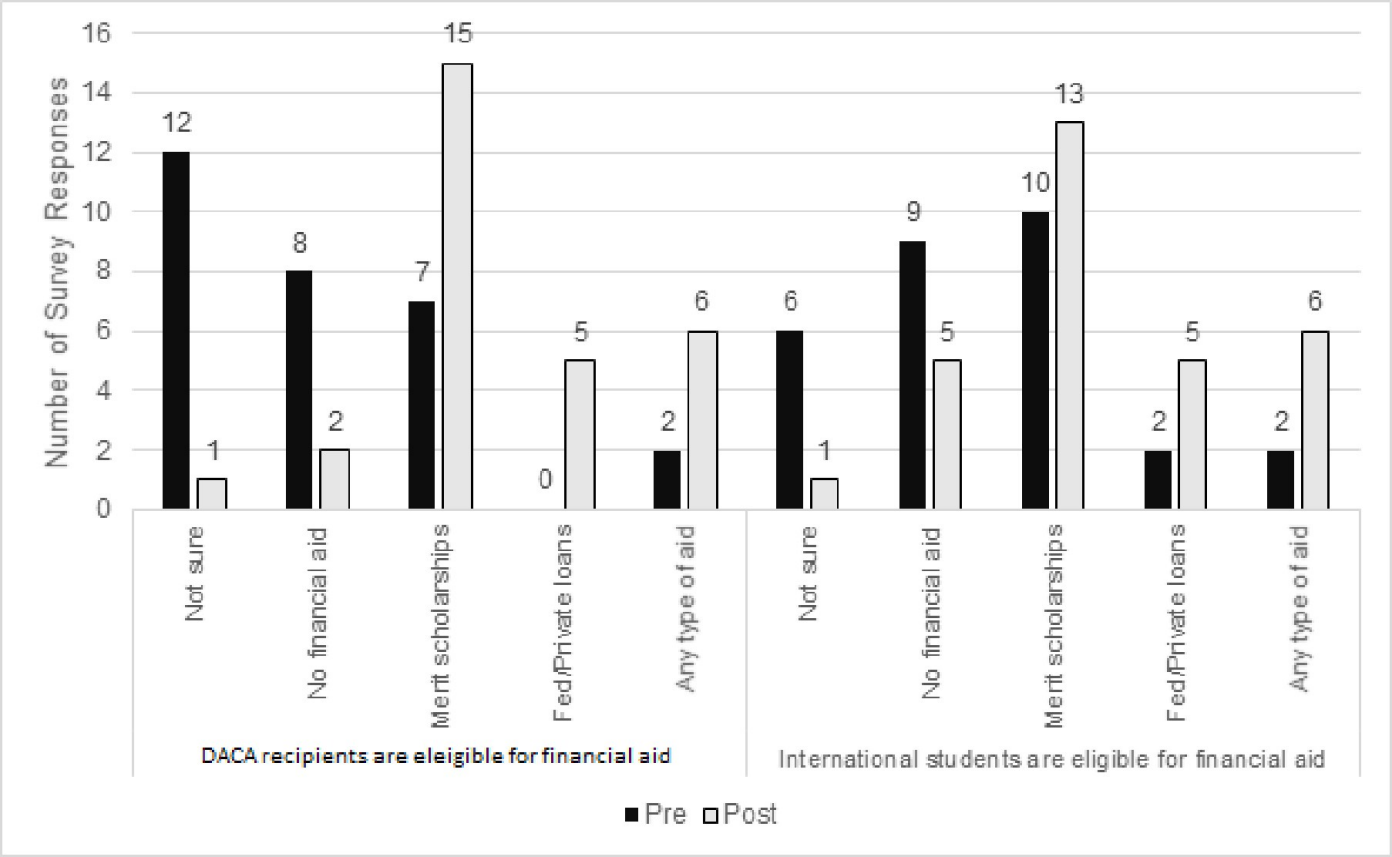
Pre-post survey results on financial aid eligibility for DACA recipients and international students. Pre-program (black) and post-program (gray) survey response comparison of participant perceptions of financial aid eligibility for students with DACA (left) and international visas (right).

**Fig 2:**
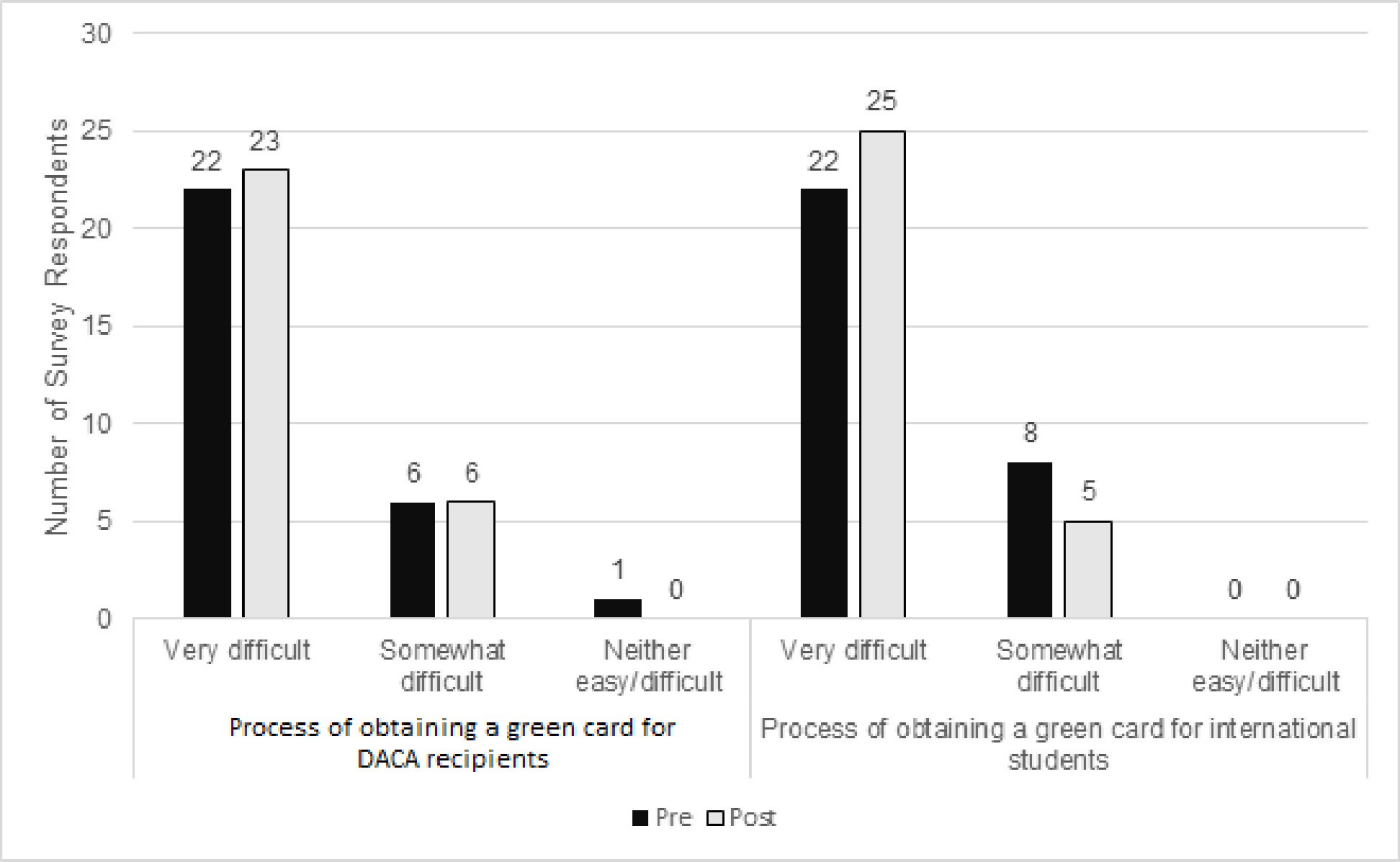
Pre-post survey results on difficulty obtaining permanent residency for DACA recipients and international students. Pre-program (black) and post-program (gray) survey response comparison of participant perceptions of difficulty obtaining permanent residency (i.e., green card) for students with DACA (left) and international visas (right).

Most changes in participant knowledge were around the ability to legally practice medicine in the U.S., eligibility for financial aid, and confidence in advising students. There was a significant change in perception for students with DACA being able to lawfully practice medicine in the U.S. pre-program (16 (53.3%) reported unsure or not eligible) compared to post-program (27 (90%) reported eligible; z = -3.13, *p = 0*.*002*; Table 2). There was no change in responses for international students lawfully practicing in the U.S. (z = -.98, *p = 0*.*327*). Prior to the program, 67% of respondents indicated that they were unsure or thought students were ineligible for any financial aid during medical school for both students with DACA (12 (40%) reported unsure, 8 (26.6%) reported not eligible) and international student visas (9 (30%) reported unsure, 9 (30%) reported not eligible). After the program, a significant proportion of respondents reported that students were eligible for merit scholarship or private loans during medical school with DACA (20 (66.6%) reported eligible, z = -3.67, *p <* .*001*) and international visas (18 (60%) reported eligible, z = -3.37, *p <* .*001*) (Fig 1). There was no change in perception of difficulty obtaining permanent residency (green card) prior to and after the program (Fig 2).

**Table 2:** Pre-post survey results following program on supporting DACA recipients and international students in medicine.

	Pre		Post			
**Question (n = 29)**	**# Yes**	**Mdn** *	**#Yes**	**Mdn** *	**Z**	**p-value**
College students who have DACA status can attend US medical school	21	2	29	2	-1.72	0.086
College students who are international (on an F1 student visa) can attend US medical schools	27	2	30	2	-1.41	0.157
College students who are DACA status can become licensed doctors and lawfully practice medicine in the US	16	3	27	3	-3.13	0.002
College students who are international status can become licensed doctors and lawfully practice medicine in the US	21	3	25	3	-0.98	0.327
	**Pre**		**Post**			
**Question (n = 29)**	**# Fairly/ Very Confident**	**Mdn** **	**# Fairly/ Very Confident**	**Mdn** **	**Z**	**p-value**
Level of confidence advising DACA students	8	3	18	2	-4.02	< .001
Level of confidence advising international students	10	2	16	3	-3.40	< .001

*Mdn: median scale score: 0 = I was not sure, 1 = No, 2 = Yes

**Mdn: median scale score: 1 = Not at all confident, 2 = Not very confident, 3 = Neutral, 4 = Fairly confident, 5 = Very confident

After participating in the program, participants indicated they were significantly more confident in advising students with DACA (z = -4.02, *p <* .*001*) and on student visas (z = -3.40, *p <* .*001*; Table 2).

### Open-ended responses

Thematic review of open-ended survey responses is summarized with illustrative quotes in Table 3. Questions on advising students with DACA after participating in the program revealed the following themes: (1) increased confidence and enthusiasm in advising students with DACA and (2) having access to more resources. Participants’ whose responses revealed increased confidence and enthusiasm were underscored by increased knowledge base and access to resources through the program. Themes on advising international students after participating in the program were: (1) increased confidence and desire to advocate and (2) increased awareness of the burden of visas. Like advising students with DACA, increased confidence appeared to build upon gaining new resources for which advisors could refer their students. Participants who reported an increased understanding of visa challenges spoke to their desire to take on visa-associated administrative duties to alleviate burden off their students.

**Table 3:** Thematic analysis of open-ended survey responses evaluating program on supporting students with DACA and international visas in medicine program, July 2021.

Question and theme	Illustrative Quote
**Question: How will your advising of DACA students change, if at all, after this program?**	
Increased confidence and enthusiasm in advising DACA students	• I feel more confident in sharing and understanding resources and showing that I am an ally. *(Pre-health advisor*, *North Carolina)* • Be more positive about chances and be encouraging that even though it is difficult it is doable. *(Pre-health advisor*, *Illinois)* • Emphasize importance of applying to DACA-friendly schools, provide guidance on how to identify those programs. Also emphasize challenges of financing education (although students already know that). Above all, encourage them to pursue their health care plans and support them however I can. *(Pre-health advisor*, *Wisconsin)*
Access to more resources	• I have a network of resources I can point them to and stories of success with which to frame the conversation. *(Pre-health advisor*, *Leadership role in college advising*, *Connecticut)* • When I asked the panelists what was the most support they received, they shared that it was working with someone with first-hand experience of the process of being DACAmented in medical school. I will keep a broad network of people I can reach out to for this kind of mentorship since I cannot offer that level of support. *(Pre-health advisor*, *North Carolina)* • I feel like I now have up-to-date materials which will better enable me to advise DACA students when they disclose their status (I always mention DACA/DREAMer in presentations but have yet to have a student share that they are undocumented at my current institution). *(Pre-health advisor*, *Iowa)*
**Question: How will your advising of international students change, if at all, after this program?**	
Increased confidence and desire to advocate for international students	• I will be able to feel more confident in assisting them to find resources and what to look forward to as they continue in their application. *(General college advisor*, *Pre-health advisor*, *Florida)* • I will be more mindful/knowledgeable to know where to find information for these students and support networks available to them. *(Pre-health advisor*, *Michigan)*
Increased awareness of the burden of visas	• In addition to pointing out the list of schools that accept international applicants on the F1docs website, I will encourage them to seek out a mentor via the website. I have referred them to our university’s international center in the past to ask questions regarding CPT, however moving forward I make more an effort to find answers for these students; this webinar helped me to realize just how much they have on their plates and how overwhelming it can be. *(Pre-health advisor*, *North Dakota)* • I will be more aware of the visa limitations, the need to save some time for residency and fellowships, and the huge amount of paperwork and navigation needed for a successful medical school training process in the US. *(Pre-health advisor*, *Leadership role in college advising*, *North Carolina)*
**Question: Please feel free to share any additional comments about the program.**	
General excitement about the program	• This was a fantastic program. I learned so much that I can share with students and resources to which I can reach out and continue to learn from. *(Pre-health advisor*, *North Carolina)* • It was honestly one of the best virtual trainings I’ve been to within the past year. Wonderfully organized, fantastic speakers, and a great mix of sessions. *(Pre-health advisor*, *Leadership role in college advising*, *Connecticut)* • I really enjoyed this program! I wish our university had a center that focused on pre-health/pre-med dreamers/DACA and international students. There’s hardly any diversity represented in the current advising office for pre-health students. I worked with a DACA pre-med student and I am certain he did not feel comfortable talking to anyone because of that reason…I definitely want to see this change in our institution so we can see our DACA, Dreamers and international students succeed and feel proud of the university they got their undergraduate degrees from. Excellent job!! *(General college advisor*, *Pre-health advisor*, *Florida)*
Value of hearing first-hand stories from panelists currently in medicine	• I loved the personal stories from the panelists. I would also love to have a document detailing the various rules about visas, work permits, and the times/deadlines for these various documents. *(Pre-health advisor*, *Leadership role in college advising*, *North Carolina)* • I enjoyed hearing the stories of undocu/DACA students, and the panel where they answer our questions. It’s very encouraging to hear these stories of success as they are a source of inspiration for future generations to come. *(Other*, *District of Columbia)*
Desire for ongoing targeted programming	• This was a very helpful program. I would love for you to be able to repeat it annually, both to refresh advisors and to help us understand potential changes in national, state, and school policies that affect intl. and DACA/Dreamer students. Programming for undergrads would be great too. *(Pre-health advisor*, *Wisconsin)*

^a^ The authors gathered all exemplary quotes from short-answer responses in the surveys. They extracted themes from the qualitative data to understand the experiences of participants and the impact the program had on them.

When asked to reflect on the program experience, the following themes emerged: (1) general excitement about the program; (2) value of hearing first-hand stories from panelists currently in medicine; and (3) desire for ongoing targeted programming. These quotes highlighted participants’ positive experiences being closely tied to the uniqueness of the program, affirmation in hearing personal stories, and desire for continuation of this curriculum.

## Discussion

To our knowledge, this is the first virtual program for health professional advisors and educators on supporting DACA recipients and international students on their medical school journey. This pilot program was well received and successfully filled some gaps in advisor knowledge regarding admissions, financial aid, and legal medical practice. The program provided concrete resources and identified misconceptions that could be hindering or further challenging students with DACA or on visas pursuing medical careers in the U.S.

A crucial element of this program was virtual instruction. Due to COVID-19, we were able to utilize an accessible, free online format that allowed advisors from all over the country to learn from scholars and hear direct experiences from DACA recipients, international students, and physicians at various levels of training. Hearing first-hand experiences is essential for demystifying stigma and inaccurate perceptions around non-citizens entering medical education and the healthcare profession. Immigrant physicians are an essential part of healthcare and supporting students earlier in their training may alleviate individual burden and promote diversity of the larger workforce [6, 9, 24].

Our study also supports previous studies which have found that fundamental immigration policy may not be known to key educators and stakeholders [21]. This pilot was focused on advisors who directly work with students entering medical education, however, even more important may be awareness of immigration policy among medical education and healthcare leadership. Institutional leaders who often make decisions about admissions, scholarship accessibility, and residency restrictions were not part of this program. Including these groups in future iterations may promote cultural competence among stakeholders and incite further change.

There are several other future directions for this pilot program. Students who are DACA recipients or on student visas were included in this program to share their personal experiences, however, were not a target audience. While we chose advisors as our participants in hopes of having a trickle-down effect and reaching greater numbers of students, it is important to make this information directly available to students who are, or will be, going through medical school and residency as non-U.S. citizens or non-green-card holders. Future programming tailored to students without immigration status, who are DACA recipients, or are on a student visa is an important consideration. Additionally, developing a similar program for medical school educators and career advisors may benefit students who are applying to residency or seeking employment [14, 25]. Further, it is unclear how our findings may translate into medical education outside the U.S. More information is needed to understand whether medical students of similar immigration status in other countries face similar challenges and, if so, how targeted programming for advisors may improve admission outcomes and student experiences.

There are areas of improvement within this pilot curriculum. We found that, even after going through the program, the proportion of respondents who reported that students were eligible for merit scholarship during medical school was still low. Some participants continued to incorrectly report that students would be eligible for any type of financial aid, including federal aid, which they are not. These findings may be partially explained by the fact that our survey response combined private and federal aid into one option, despite most students only being eligible for private aid. Information on navigating finances was dispersed throughout the program, but we did not have a specific session on financial aid which may have made the information difficult for participants to grasp. In the future, participants may benefit from a session which specifically covers financial aid for students with DACA and student visas to increase post-program knowledge.

One of our participants noted (Table 3) the potential value of having concrete documents to take away from the program with information on rules and regulations of different immigration statuses. We did not provide take-home documents from this program due to concern of maintaining updated information with frequently changing policies. Collaborating with organizations, such as PHD and F1 Doctors, to create a live and interactable document for advisors is another future direction of this program.

While DACA recipients and international students attending U.S. medical schools are small in numbers, the lack of targeted mentorship in medical school was the core impetus for this program. One consideration for future research and programming is better understanding and addressing the challenges of entering residency and the healthcare profession. Students who have DACA are often bilingual, come from underserved backgrounds, and are more likely to serve in underserved communities as practicing physicians. Thus, investing in these students may be a meaningful step for addressing health inequities [6, 26, 27].

There are several limitations in our study. First, our participant pool was small, and our survey response rates were low. We strove to overcome response bias by sending repeated reminders and offering a gift card incentive. Second, our program was held at the end of July which coincided with medical school admissions and college orientation. It is possible that participants from less resourced programs were unable to attend due to the time commitment. We tried to address this by providing recordings after the sessions (excluding panel discussions for reasons stated above) and schedule the program in the afternoon. We conducted an analysis of open-ended responses, however, were unable to conduct an in-depth qualitative analysis through methods such as interviews or focus groups. Finally, retrospective pre-post surveys are susceptible to biased reporting. We chose this format to avoid survey fatigue and to eliminate the need for linking our data with participant IDs or identifiable markers. Further studies are warranted to evaluate this program.

## Conclusions

This pilot program was an effective first step in providing targeted support for medical students who are non-citizens or permanent residents. These findings show the need for more information on advising DACA recipients and international students prior to medical school admissions, and throughout training as many educators are unaware of their unique circumstances and challenges. The structure of this virtual program can be used as a cost- and time-efficient method for connecting educators with students and physicians who have successfully navigated the intricacies of U.S. immigration and are eager to share their stories.

## Supporting information

S1 Appendix Retrospective pre-post survey evaluating program on supporting DACA recipients and international students in medicine program, July 2021. Online retrospective pre-post survey distributed to all participants.
(DOCX)
