# Supplementary material for: Supporting DACA recipients and international students pursuing careers in medicine: A pilot program for advisors
Source: PLoS One. 2023 Feb 6;18(2):e0281540. doi: 10.1371/journal.pone.0281540 (PMC9901746; doi:10.1371/journal.pone.0281540)
Supplement: S1 Appendix — Online retrospective pre-post survey distributed to all participants. (DOCX) [file pone.0281540.s001.docx]

**S1 Appendix.** **Retrospective Pre-Post Survey Evaluating Program on Supporting DACA Recipients and International Students in Medicine Program, July 2021.** Online retrospective pre-post survey distributed to all participants.

1. What is your current role in college advising? Please select all that apply.

I am a general college advisor

I am a pre-med or pre-health college advisor

I have a leadership role in college advising services (such as creating peer mentorship groups or tutoring programs)

I am a college peer advisor

Other (Please specify)

1. How many years have you been involved in college advising?
2. What kind of college are you affiliated with? Private / Public
3. In which US state do you currently advise?
4. How would you describe your school locale?

*If unsure, please see classifications here: https://nces.ed.gov/programs/edge/docs/LOCALE_CLASSIFICATIONS.pdf*

City / Suburban / Town / Rural / Unsure

1. What type of school do you advise at?

Community college

Historically Black colleges and universities (HBCU)

Liberal arts college

Women’s college

Tribal college

Technical institute and professional school

University

Other

**Please answer these next questions about what you knew BEFORE this 2-day educational program on DACA/DREAMer/International students.**

1. College students who are DACA/DREAMer status can attend US medical schools.

Yes, I thought they could apply.

No, I thought they could not apply.

I was not sure.

1. College students who are international (on an F1 student visa) can attend US medical schools.

Yes, I thought they could apply.

No, I thought they could not apply.

I was not sure.

1. College students who are DACA/DREAMer status can apply to:

No M.D. or D.O. granting medical schools in the US.

Under 10 US medical schools, including both M.D. and D.O. programs.

Under 30 US medical schools, including both M.D. and D.O. programs.

Under 50 US medical schools, including both M.D. and D.O. programs.

Under 100 US medical schools, including both M.D. and D.O. programs.

Only M.D. granting medical schools (approximately 155) in the US.

Only D.O. granting medical schools (approximately 40) in the US.

All M.D. and D.O. granting medical schools in the US.

I had no idea how many.

1. College students who are international (on a visa) can apply to:

No M.D. or D.O. granting medical schools in the US.

Under 10 US medical schools, including both M.D. and D.O. programs.

Under 30 US medical schools, including both M.D. and D.O. programs.

Under 50 US medical schools, including both M.D. and D.O. programs.

Under 100 US medical schools, including both M.D. and D.O. programs.

Only M.D. granting medical schools (approximately 155) in the US.

Only D.O. granting medical schools (approximately 40) in the US.

All M.D. and D.O. granting medical schools in the US.

I had no idea how many.

1. College students who are DACA/DREAMer status are eligible for financial aid during medical school (in the US).

Yes, I thought they were eligible for all forms of financial aid.

Yes, I thought they were eligible for only federal or private loans.

Yes, I thought they were eligible for only merit scholarships.

No, I did not think they were eligible for any financial aid.

I was not sure.

1. College students who are international (on a visa) are eligible for financial aid during medical schools (in the US).

Yes, I thought they were eligible for all forms of financial aid.

Yes, I thought they were eligible for only federal or private loans.

Yes, I thought they were eligible for only merit scholarships.

No, I did not think they were eligible for any financial aid.

I was not sure.

1. College students who are DACA/DREAMer status can become licensed doctors and lawfully practice medicine in the US.

Yes, I thought they could become licensed doctors and practice medicine in the US.

No, I thought they could not become licensed doctors and practice medicine in the US.

No, I thought they could only become licensed doctors and practice medicine in their country of origin.

I was not sure if they could become doctors and practice medicine in the US.

1. College students who are international (on a visa) can become licensed doctors and lawfully practice medicine in the US.

Yes, I thought they could become licensed doctors and practice medicine in the US.

No, I thought they could not become licensed doctors and practice medicine in the US.

No, I thought they could only become licensed doctors and practice medicine in their country of origin.

I was not sure if they could become doctors and practice medicine in the US.

1. Do you advise DACA/DREAMer status students?

Yes / No / I'm not sure

1. How confident were you in advising DACA/DREAMer students (before the program)?

Not at all confident in advising DACA/DREAMer status students.

Not very confident in advising DACA/DREAMer status students.

Neither confident or unconfident in advising DACA/DREAMer status students.

Fairly confident in advising DACA/DREAMer status students.

Very confident in advising DACA/DREAMer status students.

1. What did you think about the process of obtaining a green card (permanent residency) or US citizenship for DACA/DREAMer status students (before the program)?

Very easy

Somewhat easy

Neither easy nor difficult

Somewhat difficult

Very difficult

1. Do you advise international students?

Yes / No / I'm not sure

1. How confident were you in advising international students (before the program)?

Not at all confident in advising international students.

Not very confident in advising international students.

Neither confident or unconfident in advising international students.

Fairly confident in advising international students.

Very confident in advising international students.

1. What did you think about the process of obtaining a green card (permanent residency) or US citizenship for international students (before the program)?

Very easy

Somewhat easy

Neither easy nor difficult

Somewhat difficult

Very difficult

**Please answer these next questions about what you know AFTER this program.**

1. College students who are DACA/DREAMer status can attend US medical schools.

Yes, I think they can attend.

No, I do not think they can attend.

I am not sure.

1. College students who are international (on a student visa) can attend US medical schools.

Yes, I think they can attend.

No, I do not think they can attend.

I am not sure.

1. College students who are DACA/DREAMer status can apply to:

No M.D. or D.O. granting medical schools in the US.

Under 10 US medical schools, including both M.D. and D.O. programs.

Under 30 US medical schools, including both M.D. and D.O. programs.

Under 50 US medical schools, including both M.D. and D.O. programs.

Under 100 US medical schools, including both M.D. and D.O. programs.

Only M.D. granting medical schools (approximately 155) in the US.

Only D.O. granting medical schools (approximately 40) in the US.

All M.D. and D.O. granting medical schools in the US.

I am not sure how many.

1. College students who are international (on a visa) can apply to:

No M.D. or D.O. granting medical schools in the US.

Under 10 US medical schools, including both M.D. and D.O. programs.

Under 30 US medical schools, including both M.D. and D.O. programs.

Under 50 US medical schools, including both M.D. and D.O. programs.

Under 100 US medical schools, including both M.D. and D.O. programs.

Only M.D. granting medical schools (approximately 155) in the US.

Only D.O. granting medical schools (approximately 40) in the US.

All M.D. and D.O. granting medical schools in the US.

I am not sure how many.

1. College students who are DACA/DREAMer status are eligible for financial aid during medical schools (in the US).

Yes, I think they are eligible for all forms of financial aid.

Yes, I think they are eligible for only federal or private loans.

Yes, I think they are eligible for only merit scholarships.

No, I do not think they are eligible for any financial aid.

I am not sure.

1. College students who are international (on a visa) are eligible for financial aid during medical schools (in the US).

Yes, I think they are eligible for all forms of financial aid.

Yes, I think they are eligible for only federal or private loans.

Yes, I think they are eligible for only merit scholarships.

No, I do not think they are eligible for any financial aid.

I am not sure.

1. College students who are DACA/DREAMer status can become licensed doctors and lawfully practice medicine in the US.

Yes, I think they can become licensed doctors and practice medicine in the US.

No, I do not think they can become licensed doctors and practice medicine in the US.

No, I think they can only become licensed doctors and practice medicine in their country of origin.

I am not sure if they can become doctors and practice medicine in the US.

1. College students who are international (on a visa) can become licensed doctors and lawfully practice medicine in the US.

Yes, I think they can become licensed doctors and practice medicine in the US.

No, I do not think they can become licensed doctors and practice medicine in the US.

No, I think they can only become licensed doctors and practice medicine in their country of origin.

I am not sure if they can become doctors and practice medicine in the US.

1. How confident are you now in advising DACA/DREAMer students (after the program)?

Not at all confident in advising DACA/DREAMer status students.

Not very confident in advising DACA/DREAMer status students.

Neither confident or unconfident in advising DACA/DREAMer status students.

Fairly confident in advising DACA/DREAMer status students.

Very confident in advising DACA/DREAMer status students.

1. What did you think about the process of obtaining a green card (permanent residency) or US citizenship for DACA/DREAMer status students (after the program)?

Very easy

Somewhat easy

Neither easy nor difficult

Somewhat difficult

Very difficult

1. How confident are you now in advising international students (after the program)?

Not at all confident in advising international students.

Not very confident in advising international students.

Neither confident or unconfident in advising international students.

Fairly confident in advising international students.

Very confident in advising international students.

1. What did you think about the process of obtaining a green card (permanent residency) or US citizenship for international students (after the program)?

Very easy

Somewhat easy

Neither easy nor difficult

Somewhat difficult

Very difficult

**Please answer these next questions at whatever length you choose.**

1. How have you advised pre-med DACA/DREAMer students in the past?
2. How will your advising of DACA/DREAMer students change, if at all, after this program?
3. How have you advised pre-med international students in the past?
4. How will your advising of international students change, if at all, after this program?
5. Please feel free to share any additional comments about the program.
